# Improving Decision Making about Genetic Testing in the Clinic: An Overview of Effective Knowledge Translation Interventions

**DOI:** 10.1371/journal.pone.0150123

**Published:** 2016-03-03

**Authors:** France Légaré, Hubert Robitaille, Claire Gane, Jessica Hébert, Michel Labrecque, François Rousseau

**Affiliations:** 1 Population Health and Practice-Changing Research Group, Centre hospitalier universitaire de Québec Research Centre, Quebec, Canada; 2 Department of Family Medicine and Emergency Medicine, Laval University, Quebec, Canada; 3 Department of Molecular Biology, Medical Biochemistry and Pathology, Laval University, Quebec, Canada; 4 APOGÉE-Net/CanGèneTest Research and Knowledge Network on Health Services and Policy in Genetics and Genomics, Quebec, Canada; Ottawa Hospital Research Institute, CANADA

## Abstract

**Background:**

Knowledge translation (KT) interventions are attempts to change behavior in keeping with scientific evidence. While genetic tests are increasingly available to healthcare consumers in the clinic, evidence about their benefits is unclear and decisions about genetic testing are thus difficult for all parties.

**Objective:**

We sought to identify KT interventions that involved decisions about genetic testing in the clinical context and to assess their effectiveness for improving decision making in terms of behavior change, increased knowledge and wellbeing.

**Methods:**

We searched for trials assessing KT interventions in the context of genetic testing up to March 2014 in all systematic reviews (n = 153) published by two Cochrane review groups: Effective Practice and Organisation of Care (EPOC) and Consumers and Communication.

**Results:**

We retrieved 2473 unique trials of which we retained only 28 (1%). Two EPOC reviews yielded two trials of KT interventions: audit and feedback (n = 1) and educational outreach (n = 1). Both targeted health professionals and the KT intervention they assessed was found to be effective. Four Consumers and Communication reviews yielded 26 trials: decision aids (n = 15), communication of DNA-based disease risk estimates (n = 7), personalized risk communication (n = 3) and mobile phone messaging (n = 1). Among these, 25 trials targeted only health consumers or patients and the KT interventions were found to be effective in four trials, partly effective in seven, and ineffective in four. Lastly, only one trial targeted both physicians and patients and was found to be effective.

**Conclusions:**

More research on the effectiveness of KT interventions regarding genetic testing in the clinical context may contribute to patients making informed value-based decisions and drawing the maximum benefit from clinical applications of genetic and genomic innovations.

## Introduction

All healthcare systems are faced with the challenges of improving quality of care and contributing to population health using the best available evidence [1, 2]. Globally, health systems fail to use evidence optimally, and evidence pertaining to innovations in genetics and genomics is no exception [3, 4]. Knowledge translation (KT), also known as implementation science, is broadly defined as the method for closing the gaps between knowledge and practice [5, 6]. KT is a dynamic and iterative process [6] that seeks to mobilize best-practice evidence to guide decisions in healthcare and is an integral component of the evidence-based practice movement. KT aims to reduce the evidence-practice gap by developing, implementing, and evaluating strategies designed to enhance awareness and promote behavior change congruent with research evidence [7]. This behavior change can take place among policy makers, healthcare managers, health professionals or healthcare consumers and ultimately aims at improving the health of populations [2]. As defined by McKibbon and colleagues, KT is an umbrella term that includes “controlled trials of interventions to improve clinical performance, formal modelling of the processes involved with KT, and qualitative studies of why and how interventions worked” [8].

In the case of the biology and genetics of disease, major advances in our knowledge are fast outstripping the application of these findings to clinical contexts [3, 4, 9]. Indeed, there is little consensus not only about *how* to translate this knowledge into practice, but even about *if* its translation into practice will change behavior and/or improve the health of populations [10]. There is a gap between the availability of genetic testing and the relative importance of test results to treatment decisions [11]. In spite of heightened public expectations, there is insufficient evidence available on the validity or even the utility of genetic testing [12]. Nevertheless, fundamental research in genetics and genomics has already entered routine clinical practice, especially in the form of genetic tests for fetal abnormalities and for cancer [13, 14]. Every decision about genetic testing confronts the patient with further, increasingly difficult decisions [1, 6, 8, 15, 16]. Decisions resulting from these tests may involve preventive surgery, termination of pregnancy, or major lifestyle changes. It is therefore critical that patients be adequately informed about the risks of such tests, including the risk that taking the test will have little or no impact on their long-term health, and that they be supported in such decisions [17]. In this context, there are increasing calls for improving risk-benefit communication strategies and deliberation tools (i.e., training, feedback, education, risk counselling, or decision aids) to help patients and their healthcare providers make these increasingly complex decisions [11].

We considered that an overview of trials of KT interventions whose goal was to improve decision making about genetic testing currently practised in the clinic and the impact of these interventions on three outcomes relating to decisional quality, namely knowledge, behavior and patient wellbeing [18, 19], would contribute to the knowledge base on implementation strategies that could be relevant to decision making about genetic testing in other treatment settings [2]. It would also position the results of individual studies within an overall body of knowledge. We therefore performed an overview of trials that assessed KT implementations relevant to improved decision making about genetics in the clinical context. We explored the outcomes that authors considered measures of effectiveness, and focused on specific outcome categories that we considered relevant to improved decision making in the clinical context.

## Materials and Methods

### Data sources and search strategies

As of March 1^st^ 2014, we searched: i) the online database (library) of the Cochrane Effective Practice and Organisation of Care (EPOC) Review Group [20]; ii) the online database of the Cochrane Consumers and Communication Review Group [21]. The Cochrane Collaboration is an international not-for-profit and independent organization dedicated to making up-to-date, accurate information about the effects of healthcare interventions readily available worldwide. It produces and disseminates high quality systematic reviews of healthcare interventions and promotes the search for evidence in the form of randomized clinical trials but also of non-randomized trials, with safeguards to minimize risk of bias. In Cochrane reviews, eligibility criteria are pre-defined and typically based on types of population (participants), types of interventions (and comparisons), and types of studies that have addressed the area of interest. Occasionally reviewers restrict eligibility to specific outcomes. Studies are sought by linking together multiple reports of the same study and using this information to determine which studies are eligible for inclusion. Reviews are updated every two years. If not, a commentary is appended to explain why.

We chose the EPOC group and the Consumers and Communication Review group because both are dedicated to producing syntheses of KT intervention evaluations in the health sector. The EPOC group focuses on interventions designed to improve the delivery, practice, and organization of health care services and thus focuses on KT that targets health professionals, managers and policy makers [20]. The Consumers and Communication group coordinates the production of systematic reviews of interventions which affect consumers' interactions with healthcare professionals, services and researchers and thus focuses on knowledge translation for the public and patients [21]. Together, these review groups constitute a valuable source of evidence-based information for reviewing trials of KT interventions whose goal is to improve informed decision making regarding genetic testing in the clinical context.

We created a database (EndNote library) of all the published Cochrane reviews retrieved from the online library of the EPOC and Consumers and Communication Review groups. Studies from overviews (as opposed to systematic reviews) were excluded. After removing duplicate studies, we assessed all the remaining studies for eligibility.

### Eligibility criteria of studies

#### Type of study

We included randomized controlled trials (RCTs) and non-randomized controlled trials (NRCTs), controlled before and after studies (CBAs) and interrupted time series (ITS) analyses, as recommended by the EPOC Group. RCTs, NRCTs and CBAs had to have at least two intervention sites and two control sites to reduce the confounding influence of site-specific variables. ITS studies were excluded if they did not have a clearly defined point in time when the intervention occurred and at least three data points before and after the intervention.

#### Type of participant

There was no restriction regarding participant characteristics. Participants included: 1) healthcare professionals, including professionals in training who were responsible for patient care; 2) patients and other healthcare consumers; or 3) healthcare managers or policy makers.

#### Type of intervention

We included any kind of KT intervention that pertained to genetic testing. We defined a “KT intervention” as the process of intervening on people, groups, entities or objects in an experimental study in order to translate evidence about improved healthcare knowledge, behavior change or patient wellbeing, *i*.*e*., KT interventions that were relevant to improving decision making processes. In this review, we defined risk communication as conveying information, while counseling included emotional support as well [22]. We defined genetic/genomic testing as the analysis of DNA, proteins, or metabolites in order to predict or detect heritable diseases or disease-related mutations, genotypes, karyotypes, or phenotypes for clinical purposes.

#### Type of outcome measure

Outcomes were included if they were relevant to improved decision making about genetic testing, *i*.*e*., i) patient outcomes (*e*.*g*., satisfaction, knowledge, quality of life); ii) health professional outcomes (*e*.*g*., knowledge, attitudes, performance, clinical behavior); and iii) health system outcomes (*e*.*g*., costs).

### Study selection

A two-step process was used to screen studies for inclusion. Two reviewers independently assessed the titles alone or titles and abstracts of all primary studies collected in the EndNote library by searching for the terms genetic, genomics and elements pertaining to genetic testing. Then, the same two independent reviewers screened the full text of studies for which abstracts appeared to meet the inclusion criteria. Disagreements were resolved by discussion with a third party (FL).

### Data extraction

Two reviewers independently extracted data from included studies. Study data were extracted using a modified version of the EPOC Data Collection Checklist [23]. Data extracted were: 1) Cochrane editorial group in which the study appeared (EPOC or Consumers and Communication Groups); 2) study characteristics, including main objective of the study, study design, country of origin, language of publication, the role of genetic testing in the study (*i*.*e*., whether it played a role in the intervention, the outcome or the diagnosis), clinical context, rationale for the study and main outcome measure; 3) characteristics of the intervention, including use of a conceptual/theoretical model relevant to KT, target of the intervention (*e*.*g*., patients, health professionals, managers, policy makers), type of intervention, tool(s) used for the intervention and its clinical setting; 4) type of outcome; and 5) effectiveness of the intervention as reported by authors. For the purposes of data extraction, knowledge translation interventions were classified as effective if their impact on at least one study outcome was reported as statistically significant.

### Data analysis

#### Trial and outcome classification

We classified as “genetic intervention studies” those in which genetic testing played a role in the intervention itself, *i*.*e*. KT interventions that assessed the effect of giving feedback about a genetic test on a subject’s health-related behavior (*e*.*g*., the effect of giving a person the positive results of a GSTM gene mutation test on smoking cessation) (see S1 Fig). “Genetic outcome studies” were studies in which genetic testing played a role in the outcome, i.e. those that aimed to assess the impact of a KT intervention on: 1) patient intention to undergo genetic testing (*e*.*g*., BRCA1/2 for breast and/or ovarian cancer or prenatal screening); 2) professional aptitude or performance in explaining genetic testing to patients; 3) patient or health-related outcomes (*e*.*g*., anxiety) of people who planned to undergo genetic testing or had already undergone testing. Finally, “genetic diagnosis studies” were those that included participants who had already tested positive as carriers of a genetic disease (*e*.*g*., Down syndrome, Spina bifida) or a genetic mutation signaling risk of a disease (*e*.*g*., BRCA1/2 for breast and/or ovarian cancer) and on whom a KT intervention was tested. Outcomes were classified into the three following categories: “knowledge”, “behavior” or “wellbeing”. We classified outcomes as “knowledge-related” if they assessed participant knowledge (*e*.*g*., knowledge about breast or ovarian cancer genetics; knowledge about risks associated with a genetic test). We classified outcomes as “behavior-related” if they assessed participant behavior (*e*.*g*., intention to undergo genetic testing; quitting smoking following testing). Finally, outcomes were classified as “wellbeing-related” if they assessed participants’ emotional wellbeing (*e*.*g*., anxiety level, decisional conflict regarding genetic testing, and decision quality measures other than those measuring knowledge and behavior). Studies concerning family susceptibility to a disease were not considered unless there was a genetic component (*e*.*g*., women with family history of breast cancer, but for whom genetic testing had not confirmed a genetic predisposition). We used simple descriptive statistics to report extracted data.

#### Effectiveness of the KT interventions

We reported the effectiveness of the KT interventions according to: a) the type of outcome, b) the KT intervention studied, and c) the target of the intervention (patient or provider). If the intervention had a statistically significant impact on all assessed outcomes, it was labelled “effective.” If it had a statistically significant impact on at least one of the assessed outcomes, it was classified as “partially effective.” If an intervention had no statistically significant impact on any of the assessed outcomes, it was labelled “ineffective”. Lastly, in order to find out if the impact of KT interventions was the same in the context of genetic testing as in other contexts, we compared the effectiveness reported in each genetic study with the overall effectiveness of the intervention as reported by the Cochrane systematic review from which it was retrieved.

### Quality assessment

When available, we transcribed the assessment of risk of bias reported in the Cochrane review in which the study was found. When it was not available (only one study), one reviewer assessed the risk of bias in the included studies using the criteria applied in the other studies and outlined in the *EPOC Review Group data collection checklist* and the *Cochrane Handbook for Systematic Reviews of Interventions* (Table 1): We assessed each quality criterion as “Done”, “Not done”, or “Unclear”, then we transformed these three scores into “Low risk”, “High risk”, and “Unclear”. We used the six standard criteria suggested for all RCTs and CBA studies: 1) sequence generation; 2) allocation concealment; 3) blinding of participants, personnel and outcome assessors; 4) incomplete outcome data; 5) selective outcome reporting; and 6) other sources of bias. A single reviewer not involved in the study selection or data extraction processes assessed the risk of bias of this single study.

**Table 1:** Quality assessment of included studies.

Author year	Risk of bias for six criteria*		
	Low	Unclear	High
**Audrain 1997** [25]	0	4	2
**Bekker 2004** [32]	2	4	0
**Bjorklund 2012** [48]	2	4	0
**Bowen 2002** [29]	1	4	1
**Chao 2008** [41]	4	1	1
**Cheng 2008** [42]	3	2	1
**Green 2001** [27]	2	4	0
**Green 2004** [33]	4	2	0
**Helmes 2006** [39]	1	2	0
**Hishida 2010** [47]	2	1	3
**Hunter 2005** [37]	3	3	0
**Ito 2006** [40]	2	1	3
**Kuppermann 2009** [45]	3	3	0
**Lerman 1997** [49]	1	5	0
**Leung 2004** [34]	4	2	0
**Marteau 2004** [35]	4	1	1
**McBride 2002** [30]	2	3	1
**Miller 2005** [38]	4	2	0
**Modell 1998** [26]	4	1	1
**Sanderson 2008** [43]	3	2	1
**Schwartz 2001** [28]	3	3	0
**Schwartz 2009** [46]	3	3	0
**Skinner 2002** [31]	1	5	0
**Smith 1995** [24]	6	0	0
**van Roosmalen 2004** [36]	2	4	0
**Wakefield 2008a** [50]	3	3	0
**Wakefield 2008b** [51]	3	3	0
**Wakefield 2008c** [44]	3	3	0

* The table shows a count of how many of the six quality criteria were judged as having “low, unclear or high” risk of bias. Six quality criteria: sequence generation; allocation concealment; blinding of participants, personnel and outcome assessors; incomplete outcome date; selective outcome reporting; and other sources of bias.

## Results

### Results of the search

**Fig 1:**
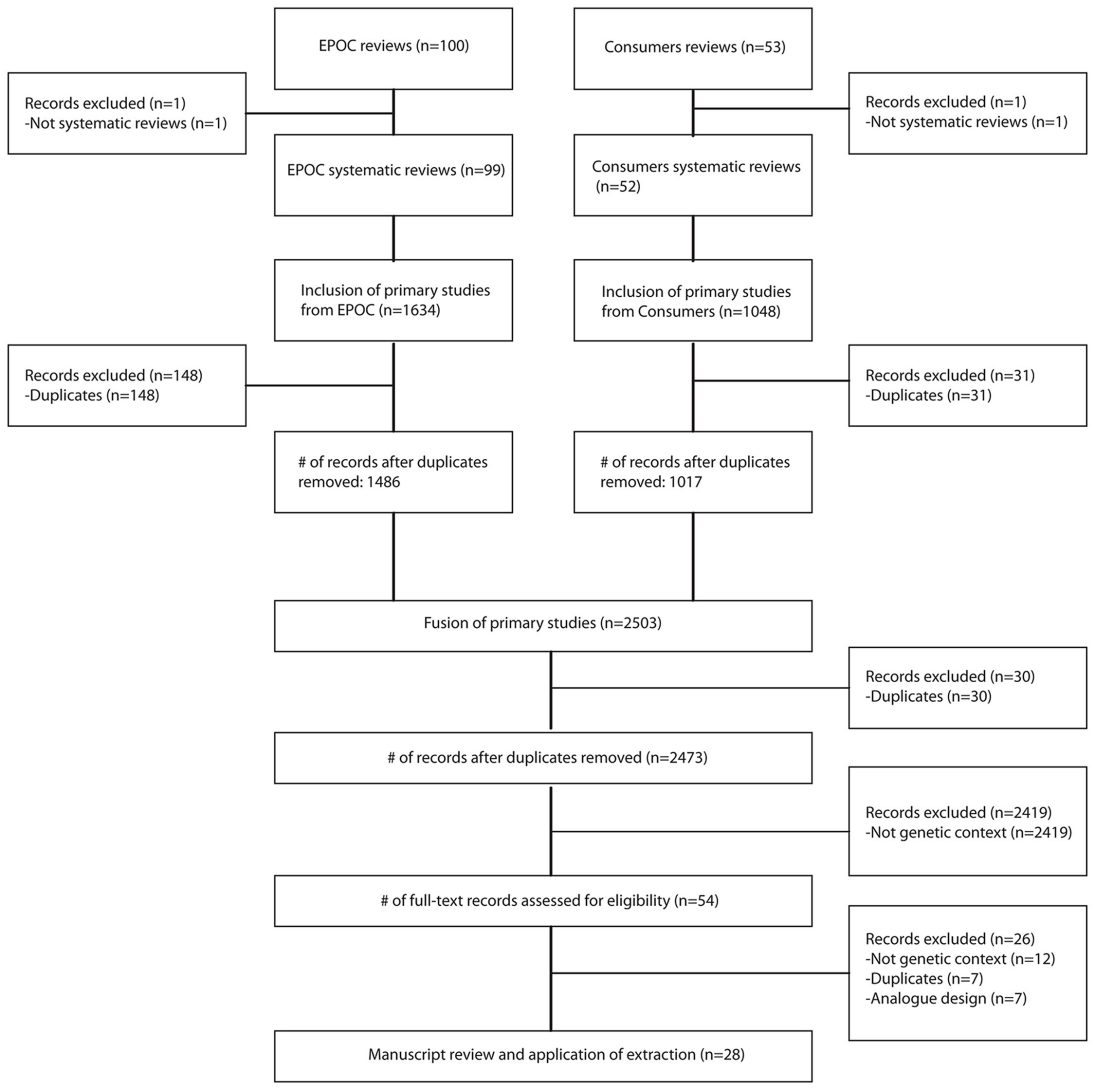
Flow diagram of study selection.

Fig 1 summarizes the flow of the search and selection processes. On March 1^st^ 2014, there were 100 published systematic reviews in the electronic EPOC Library database and 53 in the Consumers and Communication Library database. Two overviews, one in each group, were excluded. From the remaining 99 EPOC systematic reviews, 1486 primary studies were retrieved after duplicate removal (*n* = 148 duplicates; 10%), and from the 52 Consumers and Communication systematic reviews, 1017 primary studies were retrieved after duplicate removal (*n* = 31 duplicates; 3%). Once these two datasets were merged and duplicates removed (*n* = 30 duplicates; 1%), a total of 2473 primary studies were retrieved, of which 28 (1%) met our inclusion criteria [24–51]. From the 28 included studies, 26 (93%) were found in four systematic reviews [52–55] in the Consumers and Communication Library database and two studies (7%) were found in two systematic reviews [56, 57] in the EPOC Library database (see Table 2). These sources suggest that fewer KT trials in the field of genetic testing focus on health professionals and the health system than on patients and other health consumers.

**Table 2:** Study and intervention characteristics.

First author, Year*	Cochrane Group†	Authors of Cochrane review	Objective of study	Rationale	Main outcome measures	Role of genetic testing in study	Type of intervention	Tool(s) used for the intervention	Target of intervention	Clinical context	Country
**Smith, 1995 [24]**	EPOC	O’Brien, 2007 [56]	To assess 2 brief training interventions to improve obstetricians’ and midwives’ explanations to patients of a routine prenatal screening test	To improve health behavior/knowledge/wellbeing	Information-giving, communication skills and knowledge of prenatal screening	Genetic outcome (screening)	Educational outreach visits	Interpersonal, Paper, Audio/visual	Professionals(obstetricians and midwives)	Fetal anomalies	UK
**Audrain, 1997 [25]**	CCRG	Marteau, 2010 [53]	To evaluate the long-term (12 months) impact of genetic susceptibility biomarkers feedback on smoking behavior change and symptoms of depression	To improve health behavior/wellbeing	Self-reported 30-day smoking abstinence rates and depression after 12 months	Genetic intervention (prevention and genotyping)	Communicating DNA-based disease risk estimates	Interpersonal	Patient (smokers)	Lung cancer	USA
**Lerman, 1997 [49]**	CCRG	Stacey, 2014 [52]	To evaluate the impact of alternate strategies for pretest education and counseling on decision-making regarding BRCA1 testing among women at low to moderate risk who have a family history of breast and/or ovarian cancer	To increase uptake of genetic testing	Knowledge of inherited cancer and BRCA1 test characteristics, perceived risk, perceived benefits, limitations and risks of BRCA1 testing, and testing intentions at 1-month follow-up	Genetic outcome (genotyping intention)	Decision aids	Interpersonal, Paper, Audio/visual	Patient (women with family history of breast cancer)	Breast and ovarian cancer	USA
**Modell, 1998 [26]**	EPOC	Ivers, 2012 [57]	1) To investigate the feasibility of improving screening for carriers of hemoglobin disorders in general practice by using a nurse facilitator to work with primary care teams and the relevant hematology laboratories; 2) to identify problems in communication between all those involved in delivering the service, and to implement solutions	To increase uptake of genetic testing	Change in number of requests for screening tests for hemoglobin disorders	Genetic outcome (genotyping intention and diagnosis)	Audit and feedback	Interpersonal, Paper, Audio/visual	Patient and Professional (general practitioners, practice nurses)	Hemoglobin disorders	UK
**Green, 2001 [27]**	CCRG	Stacey,2014 [52]	To compare face-to-face education and counseling by a genetic counselor with education by an interactive computer program, assessing the effects of each on knowledge of breast cancer genetics and intent to undergo genetic testing	To increase uptake of genetic testing	Knowledge about breast cancer genetics and intent to undergo genetic testing at baseline and following the educational interventions	Genetic outcome (genotyping intention)	Decision aids	Interpersonal, Computer/ interactive	Patient (women with family history of breast cancer)	Breast cancer	USA
**Schwartz, 2001 [28]**	CCRG	Stacey, 2014 [52]	To examine whether a brief educational booklet regarding BRCA1/BRCA2 testing would influence knowledge, attitudes, and interest in testing among Ashkenazi Jewish women from the general population	To increase uptake of genetic testing	Knowledge and interest in BRCA 1/2 gene testing at one-month follow-up	Genetic outcome (genotyping intention)	Decision aids	Paper	Patient (Ashkenazi Jewish women from general population)	Breast and ovarian cancer	USA
**Bowen, 2002 [29]**	CCRG	Edwards, 2013 [55]	To test the effects of breast cancer counseling on interest in pursuing genetic testing in women with a family history of breast cancer	To increase uptake of genetic testing	Awareness, candidacy and interest in genetic testing at 6-month follow-up	Genetic outcome (genotyping intention)	Personalized risk communication	Interpersonal	Patient (women with family history of breast cancer)	Breast cancer	USA
**McBride, 2002 [30]**	CCRG	Marteau, 2010 [53]	To assess whether a multicomponent intervention that included feedback about genetic susceptibility to lung cancer increased risk perceptions and rates of smoking cessation compared with a standard cessation intervention	To improve health behavior/wellbeing	Self-reported as having smoked no cigarettes in the prior 7 days at the 6- and 12-month follow-ups and sustained abstinence	Genetic intervention (prevention and genotyping)	Communicating DNA-based disease risk estimates	Interpersonal, Paper	Patient(African-American smokers with low income)	Lung cancer	USA
**Skinner, 2002 [31]**	CCRG	Edwards, 2013 [55]	To compare tailored print material vs. non-tailored print material to facilitate informed genetic testing decisions	To increase uptake of genetic testing	Knowledge about genetic testing, perceived risk, expert risk estimate, accuracy of perceived risk, worries about being a mutation carrier and testing intention	Genetic outcome (genotyping intention)	Personalized risk communication	Paper	Patient (women with personal history of breast or ovarian cancer)	Breast and ovarian cancer	USA
**Bekker, 2004 [32]**	CCRG	Stacey, 2014 [52]	To evaluate decision analysis as a technique to facilitate women’s decision-making about prenatal diagnosis for Down syndrome using measures of effective decision-making	To improve health behavior/wellbeing	Test decision, subjective expected utilities, knowledge, informed decision-making, risk perception, decisional conflict, anxiety, perceived usefulness and directiveness of consultation information one month after receipt of a diagnosis test and/or the 19-week scan result	Genetic outcome (screening)	Decision aids	Paper	Patient (women receiving a screen-positive maternal serum screening test for Down syndrome)	Fetal anomalies	UK
**Green, 2004 [33]**	CCRG	Stacey, 2014 [52]	To compare the effectiveness of a computer-based decision aid with standard genetic counseling for educating women about BRCA1 and BRCA2 genetic testing	To improve health behavior/wellbeing and to increase uptake of genetic testing	Participants’ knowledge, risk perception, intention to undergo genetic testing, testing decision (at 1 and 6 months), decisional conflict, satisfaction with decision, anxiety, and satisfaction with the intervention after the counseling	Genetic outcome (genotyping intention)	Decision aids	Interpersonal, Computer/ interactive	Patient (women with personal or familial history of breast cancer)	Breast cancer	USA
**Leung, 2004 [34]**	CCRG	Stacey, 2014 [52]	1) To compare an interactive multimedia decision aid with a leaflet and a video to give information about prenatal screening for Down syndrome and 2) to determine the women’s acceptance of interactive multimedia decision aid	To improve health behavior /wellbeing and to increase uptake of genetic testing	Women’s final uptake of the screening test (integrated or serum screening) for Down syndrome and women’s initial decision, understanding and satisfaction with the information that they had received	Genetic outcome (screening)	Decision aids	Audio/visual, Computer/ interactive	Patient (pregnant women considering whether to undergo prenatal screening for Down syndrome)	Fetal anomalies	China
**Marteau, 2004 [35]**	CCRG	Marteau, 2010 [53]	To investigate the psychological impact of using genetic testing to make or confirm a clinical diagnosis	To improve health behavior/wellbeing	Perception of control over family hypercholesterolemia, cholesterol, heart disease, and fatalism about family hypercholesterolemia	Genetic intervention (prevention and genotyping)	Communicating DNA-based disease risk estimates	Interpersonal	Patient (adults clinically diagnosed with definite or possible heterozygous familial hypercholesterolemia)	Familial hypercholesterolemia	UK
**van Roosmalen, 2004 [36]**	CCRG	Stacey, 2014 [52]	To evaluate shared decision-making information for BRCA1/2 mutation carriers who have to make a choice between screening and prophylactic surgery for breast and/or ovaries	To improve health behavior/wellbeing	Well-being treatment choice and decision-related outcomes 3 and 9 months after the test result	Genetic diagnosis (mastectomy intention, as an indirect measure of the use of a DA)	Decision aids	Interpersonal, paper, audio/visual	Patient (women considering to undergo genetic testing for breast cancer)	Breast and ovarian cancer	The Netherlands
**Hunter, 2005 [37]**	CCRG	Stacey, 2014 [52]	To compare which counseling methods (individual vs. group vs. use of a decision aid) are effective in prenatal diagnosis counseling for women of advanced maternal age (≥ 35 years) and their partners	To improve health behavior/wellbeing	Knowledge, decisional conflict, state of anxiety, satisfaction, use of prenatal diagnosis, and pregnancy outcomes	Genetic outcome (screening intention, as an indirect measure of the use of a DA)	Decision aids	Interpersonal, Audio/visual	Patient (advanced age women referred for prenatal screening)	Fetal anomalies	Canada
**Miller, 2005 [38]**	CCRG	Stacey, 2014 [52]	To evaluate a novel, theory-based approach to help guide women in making informed decisions about pursuing breast cancer genetic testing	To increase uptake of genetic testing	Intention to obtain genetic testing, knowledge, perceived risk at 2-week, 2-month and 6-month follow-up	Genetic outcome (genotyping intention)	Decision aids	Interpersonal, Paper	Patient (women calling the NCI’s Cancer Information Service)	Breast cancer	USA
**Helmes, 2006 [39]**	CCRG	Edwards, 2013 [55]	To assess the effect of interventions (in person vs. by telephone vs. control) on the interest in breast cancer risk counseling and genetic testing in women at all risk levels	To improve health behavior/wellbeing and decrease uptake of genetic testing	Women’s cancer worry, risk perceptions, and intentions to obtain breast cancer screening and interest in pursuing genetic testing at 3-months follow-up	Genetic outcome (genotyping intention)	Personalized risk communication	Interpersonal, audio/visual	Patient (women of general population)	Breast cancer	USA
**Ito, 2006 [40]**	CCRG	Marteau, 2010 [53]	To evaluate whether feedback of genetic information regarding an L-myc polymorphism, identified as impacting on tobacco-related cancer risk, has an influence on smoking cessation	To improve health behavior/wellbeing	Smoking cessation at 3- and 9-months follow-ups	Genetic intervention (prevention and genotyping)	Communicating DNA-based disease risk estimates	Interpersonal, paper	Patient (smokers)	Lung cancer	Japan
**Chao, 2008 [41]**	CCRG	Marteau, 2010 [53]	To examine whether apolipoprotein E genotype and numerical risk estimate disclosure to asymptomatic individuals at high risk for Alzheimer disease alters health behaviors	To improve health behavior /wellbeing	Self-reported Alzheimer disease-specific health behavior change (changes in diet, in exercise and in medications and/or vitamins) 1 year after disclosure	Genetic intervention (prevention and genotyping)	Communicating DNA-based disease risk estimates	Paper	Patient (relative or person living with person affected by Alzheimer disease)	Alzheimer disease	USA
**Cheng, 2008 [42]**	CCRG	Gurol-Urganci, 2012 [54]	To study the effect of fast reporting by mobile phone short-message service (SMS) on anxiety levels in women undergoing prenatal biochemical screening for Down syndrome	To improve health behavior/wellbeing	Anxiety levels before prenatal screen testing, before the appointed clinic and 3 days after the appointed clinic	Genetic outcome (screening)	Mobile phone messaging	Computer/ interactive, Interpersonal	Patient (pregnant women undergoing prenatal screening for Down’s syndrome)	Fetal anomalies	Taiwan
**Sanderson, 2008 [43]**	CCRG	Marteau, 2010 [53]	To explore the impact of GSTM1 genetic testing on motivation to quit smoking	To improve health behavior/wellbeing	Intention and motivation to quit smoking within the next 6 months, depression, anxiety, perceived risk of lung cancer and comprehension of genetic test results at one-week follow-up	Genetic intervention (prevention and genotyping)	Communicating DNA-based disease risk estimates	Interpersonal, Paper	Patient (smokers)	Lung cancer	UK
**Wakefield, 2008a [50]**	CCRG	Stacey, 2014 [52]	To measure the effectiveness of a tailored decision aid designed specifically to assist individuals to make informed decisions regarding genetic testing for hereditary nonpolyposis colorectal cancer	To improve health behavior/wellbeing	Reading the materials, decisional conflict, knowledge of genetic testing, multidimensional measure of informed choice, family involvement, impact of event, hospital anxiety and depression, genetic testing decision and decision regret	Genetic outcome (genotyping intention, as an indirect measure of the use of a DA)	Decision aids	Paper	Patient (persons considering undergoing testing for colorectal cancer)	Colorectal cancer	Australia
**Wakefield, 2008b [51]**	CCRG	Stacey, 2014 [52]	To measure the effectiveness of a tailored decision aid designed to help women make informed decisions about genetic testing for breast/ovarian cancer risk	To improve health behavior/wellbeing	Reading the materials, decisional conflict, knowledge of genetic testing, multidimensional measure of informed choice, family involvement, impact of event, hospital anxiety and depression, genetic testing decision and decision regret	Genetic outcome (genotyping intention, as an indirect measure of the use of a DA)	Decision aids	Paper	Patient (women considering undergoing testing for breast cancer)	Breast and ovarian cancer	Australia
**Wakefield, 2008c [44]**	CCRG	Stacey, 2014 [52]	To evaluate the impact of a decision aid for women considering genetic testing for breast/ovarian cancer risk given during genetic counseling	To improve health behavior/wellbeing	Reading the materials, decisional conflict, knowledge of genetic testing, multidimensional measure of informed choice, family involvement, impact of event, hospital anxiety and depression, genetic testing decision and decision regret	Genetic outcome (genotyping intention, as an indirect measure of the use of a DA)	Decision aids	Paper	Patient (women considering undergoing testing for breast cancer)	Breast and ovarian cancer	Australia
**Kuppermann, 2009 [45]**	CCRG	Stacey, 2014 [52]	To estimate the effect of a computerized, interactive prenatal testing decision tool on prenatal testing decision making	To improve health behavior/wellbeing	Knowledge, risk awareness, intervention satisfaction, decisional conflict, and use of invasive diagnostic testing	Genetic outcome (screening intention, as an indirect measure of the use of a DA)	Decision aids	Computer/interactive	Patient (pregnant women)	Fetal anomalies	USA
**Schwartz, 2009 [46]**	CCRG	Stacey, 2014 [52]	To test a computer-based interactive decision aid designed to help BRCA1/2 mutation carriers make decisions about risk reducing mastectomy	To improve health behavior/wellbeing	Final management decision, decisional conflict, decisional satisfaction and receipt of risk reduction at 1-, 6- and 12- months post randomization	Genetic diagnosis (mastectomy intention, as an indirect measure of the use of a DA)	Decision aids	Computer/interactive	Patient (women who carry BRCA1/BRCA2 mutation)	Breast and ovarian cancer	USA
**Hishida, 2010 [47]**	CCRG	Marteau, 2010 [53]	To examine the effects of genotype notification of an oncogene (Lmyc) genotype to smokers on their smoking cessation behavior	To improve health behavior/wellbeing	Self-reported smoking cessation rate 1 year after the enrollment	Genetic intervention (prevention and genotyping)	Communicating DNA-based disease risk estimates	Paper	Patient (smokers)	Lung cancer	Japan
**Bjorklund, 2012 [48]**	CCRG	Stacey, 2014 [52]	To evaluate the effects of an information film on making an informed choice regarding Down syndrome screening and women’s knowledge and experience of information	To improve health behavior/wellbeing and to increase uptake of genetic testing	Informed choice (attitudes towards Down syndrome screening, knowledge about Down syndrome and Down syndrome screening and uptake of combined ultrasound biochemical screening) at week 27	Genetic outcome (screening)	Decision aids	Interpersonal, Paper, Audio/visual	Patient (pregnant women considering undergoing prenatal testing)	Fetal anomalies	Sweden

* Studies are presented in chronological order (1995–2012).

^†^ Cochrane Groups: EPOC: Effective Practice and Organisation of Care Group; CCRG: Consumers and Communication Review Group.

### Characteristics of included studies

Table 2 summarizes characteristics of the 28 studies included. In order of frequency, included studies came from the following systematic reviews retrieved in the Consumers and Communication Group: a review by Stacey (2014) on the effects of patient decision aids [52] (n = 15) [27, 28, 32–34, 36–38, 44–46, 48–51]; a review by Marteau (2010) on the effects of communicating DNA-based disease risk estimates [53] (n = 7) [25, 30, 35, 40, 41, 43, 47]; a review by Edwards (2013) on the effects of personalized risk communication [55] (n = 3) [29, 31, 39]; and a review by Gurol-Urganci (2012) on the effects of mobile phone messaging [54] (n = 1) [42]. The remaining two studies, in the EPOC Group, were retrieved from systematic reviews by Ivers (2012) on the effects of audit and feedback [57] (n = 1) [26] and by O’Brien (2007) on the effects of educational outreach visits [56] (n = 1) [24]. Eligible studies, all randomized clinical trials, were published between 1995 and 2012. Studies were conducted in the following countries in order of frequency: United States (*n* = 13) [25, 27–31, 33, 38, 39, 41, 45, 46, 49], United Kingdom (*n* = 5) [24, 26, 32, 35, 43], Australia (*n* = 3) [44, 50, 51], Japan (*n* = 2) [40, 47], The Netherlands (*n* = 1) [36], Canada (*n* = 1) [37], China (*n* = 1) [34], Sweden (*n* = 1) [48] and Taiwan (*n* = 1) [42]. All studies were published in English.

In terms of the role of genetic testing in the studies, two of the 28 studies were “genetic diagnosis studies”, i.e., genetic testing played a role in the diagnosis of a disease or a disease-related mutation (both were about mastectomy intention among women who were carrying the BRCA1/2 mutation) [36, 46]. Seven studies were “genetic intervention studies”, i.e. the intervention itself included a genetic test [25, 30, 35, 40, 41, 43, 47]. In one of these the focus was on genotyping of disease (*e*.*g*., Alzheimer disease) (n = 1) [41], in another on familial hypercholesterolemia (n = 1) [35], and in the other five, on lung cancer (n = 5) [25, 30, 40, 43, 47]. Nineteen were “genetic outcome studies”, i.e., intention to take or actually taking the test was a study outcome [24, 26–29, 31–34, 37–39, 42, 44, 45, 48–51]. Of these, 12 were about the intention to undergo genotyping [25–29, 31, 33, 38, 39, 44, 49, 50], either for breast and/or ovarian cancer (n = 10) [27–29, 31, 33, 38, 39, 44, 49, 51], colorectal cancer (n = 1) [50] or hemoglobin disorders (n = 1) [26]; and seven were about screening for fetal anomalies [24, 32, 34, 37, 42, 45, 48]. Of the 28 studies, 17 studies aimed to improve health behavior/status, seven studies aimed to increase the uptake of genetic testing and four aimed for both.

### Characteristics of interventions

#### References to conceptual models relevant to KT

Of the 28 studies, ten (36%) [24, 25, 31–33, 38, 43, 46, 47, 49] conducted a KT intervention based on or referring to a conceptual model appropriate for KT. Out of these, nine concluded that the KT intervention was effective or partly effective [24, 31–33, 38, 43, 46, 47, 49].

#### Target of the intervention

The intervention targeted only the patient in 26 studies (93%) [25, 27–51], only the health professional in one study (4%) [24] and both patient and health professional in one study (4%) [26].

#### Type of intervention

Among the 26 studies targeting patients, the KT intervention that was assessed was: decision aids [52] (n = 15) [27, 28, 32–34, 36–38, 44–46, 48–51], communicating DNA-based disease risk estimates [53] (n = 7) [25, 30, 35, 40, 41, 43, 47], personalized risk communication [55] (n = 3) [29, 31, 39], and mobile phone messaging [54] (n = 1) [42]. For the study that targeted both patient and health professional, the intervention was comprised of a decision aid for patients and audit and feedback for physicians [26]; and for the study that targeted professionals only, the intervention was educational outreach [24]. The tools used for the KT intervention were (not mutually exclusive): audio/visual materials (*n* = 8; 29%) [24, 26, 34, 36, 37, 39, 48, 49] computer or interactive material (*n* = 6; 21%) [27, 33, 34, 42, 45, 46], interpersonal intervention (*n* = 17; 61%) [24–27, 29, 30, 33, 35–40, 42, 43, 48, 49], and paper-based material (*n* = 18; 64%) [24, 26, 28, 30–32, 36–38, 40, 41, 43, 44, 47–49, 51].

#### Type of outcome

Of the 26 studies whose intervention targeted patients only, 17 (65%) studies reported knowledge-related outcomes [27–33, 36–39, 43–45, 48, 49, 51], 21 (81%) reported behavior-related outcomes [24–30, 32, 34–36, 38–41, 43, 44, 46–48, 51] and 13 (50%) reported wellbeing outcomes [30–33, 36, 37, 39, 42–46, 51] (categories not mutually exclusive). The study that targeted only professionals reported behavior-related outcomes [24]. The study that targeted both professionals and patients reported behavior-related outcomes for both health professionals and patients [26].

#### Effectiveness of the intervention

The effectiveness of the KT interventions (Table 3) are reported according to: a) the type of outcome (*i*.*e*., knowledge-related outcomes, behavior-related outcomes and wellbeing-related outcomes), b) the KT intervention studied (*i*.*e*., educational outreach to health professionals, communicating DNA-based disease risk estimates, decision aids, audit and feedback, personalized risk communication and mobile phone messaging), and c) the target of the intervention (patient or provider), both as reported by the trial authors and by the systematic review. If an intervention had a statistically significant impact on at least one outcome-type, it was classified as “effective.” In the 15 studies on decision aids, authors found them effective regarding patients’ knowledge-related outcomes in nine studies [27, 28, 32, 33, 37, 44, 45, 49–51], effective regarding wellbeing-related outcomes in nine studies [32, 33, 36, 37, 44–46, 50, 58], but effective regarding behavior-related outcomes in only six studies [28, 32, 36, 37, 45, 49] (categories not mutually exclusive). In the seven studies on communicating DNA-based disease risk estimates, authors found it effective regarding patients’ behavior-related outcomes in six studies [25, 30, 35, 40, 41, 43]. In all three studies on personalized risk communication, authors found it effective regarding patients’ knowledge-related outcomes [29, 31, 39]. The one study in which an intervention on patient *and* provider outcomes was assessed reported that the intervention was effective regarding a behavior-related outcome [26]. Overall, of the 28 included studies, 14 (50%) reported effectiveness regarding knowledge-related outcomes[24, 27–29, 31–33, 37, 39, 44, 45, 49–51], 15 (54%) reported effectiveness regarding behavior-related outcomes [24–27, 29, 30, 33, 35, 36, 38–41, 43, 46] and 13 (46%) reported effectiveness regarding wellbeing-related outcomes [25, 32, 33, 36, 37, 39, 42–46, 50, 51]. Overall, for studies targeting patients only, 12 reported a statistically significant effect on all outcomes (effective) [25, 27, 29, 30, 33, 35, 36, 39–42, 46], 11 reported both a statistically significant effect on some outcomes and none on other outcomes (partly effective) [28, 31, 32, 37, 38, 43–45, 49–51] and three reported no statistically significant effect on any outcome (ineffective) [34, 47, 48]. Authors of both studies targeting health professionals found their interventions to be effective. Only one study assessed cost-effectiveness [30].

**Table 3:** Effectiveness of interventions on decision-making outcomes.

First Author, Year*	Cost effectiveness†	Knowledge translation intervention	Effectiveness of the knowledge translation intervention as reported by authors of the primary study §			Results of trial as reported in the systematic review (intervention effective/not effective)			Do trial results match results of systematic review?
			*Knowledge related-outcomes*	*Behavior-related outcomes*	*Wellbeing-related outcomes*	*Knowledge-related outcomes*	*Behavior-related outcomes*	*Wellbeing-related outcomes*	
**Smith, 1995 [24]**	N/A	Educational outreach to health professionals	Effective for provider’s knowledge(+ at 3 months)	Effective for providers’ information-giving & communication skills (+ immediately after the intervention)			Effective		Yes
**Audrain, 1997 [25]**	N/A	Communicating DNA-based disease risk estimates		Effective for quitting smoking attempt (+ at 12 months)	Effective for depression (- at 2 months)		Not effective		No
**Lerman, 1997 [49]**	N/A	Decision Aids	Effective for knowledge (+ at 1 month)	Not effective		Effective	Not effective	Effective	Yes
**Modell, 1998 [26]**	N/A	Audit and feedback		Effective for number of screening test requests (+ at 1 year)			Effective		Yes
**Green, 2001 [27]**	N/A	Decision Aids	Effective for knowledge (+ before and after intervention	Effective for intent to undergo testing (+ before and after the intervention)		Effective	Not effective	Effective	No
**Schwartz, 2001 [28]**	N/A	Decision Aids	Effective for knowledge (+ at 1 month)	Not effective		Effective	Not effective	Effective	Yes
**Bowen, 2002 [29]**	N/A	Personalized risk communication	Effective for awareness (+ at 6 months)	Effective for interest in genetic testing and candidacy for testing (+ at 6 months)		Effective			Yes
**McBride, 2002 [30]**	Not effective	Communicating DNA-based disease risk estimates		Effective for smoking cessation (+ at 6 months)			Not effective		No
**Skinner, 2002 [31]**	N/A	Personalized risk communication	Effective for knowledge about genetic testing (+ at 2 weeks)	Not effective	Not effective	Effective			Yes
**Bekker, 2004 [32]**	N/A	Decision Aids	Effective for risk perception (+ at 1 month)	Not effective	Effective for decisional conflict (+ at 1 month)	Effective	Not effective	Effective	Yes
**Green, 2004 [33]**	N/A	Decision Aids	Effective for knowledge and perceived risk (+ immediately after the intervention)	Effective for intention to undergo genetic testing (+ immediately after the intervention) *for low risk	Effective for anxiety (+ immediately after the intervention)	Effective	Not effective	Effective	No
**Leung, 2004 [34]**	N/A	Decision Aids		Not effective		Effective	Not effective	Effective	Yes
**Marteau, 2004 [35]**	N/A	Communicating DNA-based disease risk estimates		Effective for perceived control (+ at 1 week)			Not effective		No
**van Roosmalen, 2004 [36]**	N/A	Decision Aids		Effective for decision-related outcomes (+ at 9 months); *Provider*: Effective for decision related-outcomes (+ at 9 months, observed by patients)	Effective for well-being (+ at 9 months, but not effective at 3 months)	Effective	Not effective	Effective	No
**Hunter, 2005 [37]**	N/A	Decision Aids	Effective for knowledge (+ at post-counseling)	Not effective	Effective for decisional conflict and satisfaction (+ at post-counseling)	Effective	Not effective	Effective	Yes
**Miller, 2005 [38]**	N/A	Decision Aids	Not effective	Effective for intention to obtain genetic testing (+ at 6 months)		Effective	Not effective	Effective	No
**Helmes, 2006 [39]**	N/A	Personalized risk communication	Effective for risk perceptions (+ at 3 months)	Effective for interest in genetic testing (+ at 3 months)	Effective for cancer worry (+ at 3 months)				Yes
**Ito, 2006 [40]**	N/A	Communicating DNA-based disease risk estimates		Effective for smoking cessation (+ at 9 months)			Not effective		No
**Chao, 2008 [41]**	N/A	Communicating DNA-based disease risk estimates		Effective for health behavior specific to Alzheimer disease prevention (+ at 12 months)			Not effective		No
**Cheng, 2008 [42]**	N/A	Mobile phone messaging			Effective for anxiety (+ before the appointed clinic)		Effective		N/A
**Sanderson, 2008 [43]**	N/A	Communicating DNA-based disease risk estimates	Not effective	Effective for cigarettes smoked per day and motivation to quit smoking (+ at 1 week)	Effective for depression (+ at 1 week)		Not effective		No
**Wakefield, 2008a [50]**	N/A	Decision Aids	Effective for knowledge score and informed choice (+ at 1 week)	Not effective	Effective for decisional conflict (+ at 1 week)	Effective	Not effective	Effective	Yes
**Wakefield, 2008b [51]**	N/A	Decision Aids	Effective for knowledge score (+ at 1 week)	Not effective	Effective for decisional conflict (+ at 1 week)	Effective	Not effective	Effective	Yes
**Wakefield, 2008c [44]**	N/A	Decision Aids	Effective for knowledge (+ immediately after the intervention)	Not effective	Effective for decisional conflict (+ after the intervention)	Effective	Not effective	Effective	Yes
**Kuppermann, 2009 [45]**	N/A	Decision Aids	Effective for knowledge (+ immediately after the intervention)	Not effective	Effective for intervention satisfaction and decisional conflict (+ immediately after the intervention)	Effective	Not effective	Effective	Yes
**Schwartz, 2009 [46]**	N/A	Decision Aids		Effective for management decision (+ longitudinal impact at 1, 6 and 12 months)	Effective for decisional conflict (+ longitudinal impact at 1, 6 and 12 months)	Effective	Not effective	Effective	No
**Hishida, 2010 [47]**	N/A	Communicating DNA-based disease risk estimates		Not effective			Not effective		Yes
**Bjorklund, 2012 [48]**	N/A	Decision Aids		Not effective		Effective	Not effective	Effective	No

* Studies are presented in chronological order (1995–2012).

†N/A: not applicable (outcome either not assessed, or because the outcomes assessed were not the same).

§ Effectiveness statistically significant (p≤0.05) or not statistically significantly (p>0.05).

In order to explore the performance of KT interventions in genetics compared to their performance in other domains, we looked at congruence between effectiveness of implementations as reported in the genetics studies and their effectiveness as reported in the overall review in which they were found. There were 44 cases where an outcome-type in a genetics study could be matched with an outcome-type in the review in which it was found. Of these, there were 20 (45%) in which both the genetics study and the overall review found an effect, 11 (25%) in which neither the genetics study nor the overall review found an effect, ten (22%) in which the genetics study found an effect while the overall study did not, and one (.02%) in which the genetics study found no effect, but the overall review did.

Of the 28 included studies, the conclusions of 15 were congruent with the conclusions of the Cochrane systematic review in which they were included [24, 26, 28, 29, 31, 32, 34, 37, 39, 44, 45, 47, 49–51]. Twelve contradicted the conclusion of their respective Cochrane reviews [25, 27, 30, 33, 35, 36, 38, 40, 41, 43, 46, 48] and one study did not assess the same outcomes [42] (Table 3).

Regarding the effectiveness of the intervention type in studies that assessed any outcome, decision aids were significantly effective in 68% (23/34) of the studies, communicating DNA-based disease risk estimates interventions was significantly effective in 80% (8/10) of the studies, audit and feedback was significantly effective in 100% (1/1) of the studies, personalized risk communication was significantly effective in 75% (6/8) of the studies, mobile phone messaging was significantly effective in 100% (1/1) of the studies and educational outreach to health professionals was significantly effective in 100% (2/2) of the studies (Table 4).

**Table 4:** Effectiveness of each type of intervention on assessed outcomes types.*

Intervention type	Studies reporting effectiveness on outcome-type A / total studies reporting on outcome-type A	Studies reporting effectiveness on outcome-type B / total studies reporting on outcome-type B	Studies reporting effectiveness on outcome-type C / total studies reporting on outcome-type C
**Decision aids (N = 15)**	9/10	5/15	9/9
**Communicating DNA-based disease risk estimates (N = 7)**	0/1	6/7	2/2
**Audit and feedback (N = 1)**	0/0	1/1	0/0
**Personalized risk communication (N = 3)**	3/3	2/3	1/2
**Mobile phone messaging (N = 1)**	0/0	0/0	1/1
**Educational outreach to health professionals (N = 1)**	1/1	1/1	0/0

*Fractions are number of studies reporting effectiveness on an outcome-type / total number of studies that assessed that outcome-type (not mutually exclusive). A = knowledge-related outcomes; B = behavior-related outcomes; C = wellbeing-related outcomes; N = total number of studies that assessed this type of intervention.

### Risk of bias in included studies

Among the 28 included studies, eight studies scored a 1 or 2 high risk of bias and two scored a 3 high risk of bias. None scored more than 3 high risk of bias. This suggested low risk of bias in most studies. The risk of bias across studies is summarized in Table 1.

## Discussion

Out of 2473 unique studies obtained from 151 systematic reviews published by two Cochrane editorial groups (those dedicated to producing syntheses of KT intervention evaluations in the health sector), we identified only 28 (1%) trials that were informative about the effectiveness of KT interventions regarding genetic testing. These were produced mainly in the USA and the UK and all were randomized trials, which is the most robust study design for assessing the effect of an intervention [59, 60]. Overall, the included trials were found to be at low risk of bias. Clinical domains most often addressed were breast and/or ovarian cancer, lung cancer and prenatal screening for fetal anomalies. Only two of these trials targeted health professionals: one used educational outreach visits and the other, audit and feedback combined with decision aids for patients. In both trials, the KT intervention was found to be effective. All remaining trials (n = 26) targeted only patients/health consumers, with the majority assessing decision aids, followed by communication of DNA-based disease risk estimates, personalized risk communication and mobile phone messaging. In the 15 trials that assessed the effect of decision aids on patients, 87% reported a statistically significant effect, while the seven trials that assessed communicating DNA-based disease risk estimates all reported a significant effect at different levels. In 70% of the cases where outcome types reported in a genetic study matched those reported in the review from which it was retrieved, there was congruence between the effectiveness or not of the interventions. Based on our findings, our main take-home messages are the following: there are very few trials that rigorously evaluate KT interventions in the field of genetic testing; little is known about their effectiveness in the field of genetic testing outside of the USA and the UK; little is known about their effectiveness in clinical contexts other than that of breast and/or ovarian cancer, lung cancer and prenatal screening; some knowledge is available about the effectiveness of KT interventions in the field of genetic testing that target patients, such as decision aids, but little is known about those that target health care professionals; and rigorous evaluations of the various components of KT interventions are needed for a better understanding of what could make them more effective. Our results lead us to make four further observations.

First, it is not surprising to find so few trials of KT interventions regarding genetic testing. This paucity of evidence may be because the usefulness of much genetic testing is still unclear. Our review showed that relevant clinical applications of knowledge about genetic predispositions are still rare except in the areas of cancer care and prenatal screening. Even among the few relevant trials we were able to retrieve, some studies show that providing genetic risk information will not, long-term, contribute to changing people’s risky behavior, e.g., help them to stop smoking [25]. Others point to unintended consequences of genetic testing, such as Marteau's study indicating that a DNA-based diagnosis of familial hypercholesterolemia may reduce confidence in dietary interventions [35]. Yet others focus on testing that is widely considered to lack clinical utility (APOE testing to assess Alzheimer risk) [41] and in one of the studies, breast cancer interventions achieved only limited success in reducing interest in genetic testing among lower-risk women for whom genetic testing was not recommended [29]. The 70% of cases in which the results of the genetics study and of the overall review were congruent provides some evidence that KT interventions in other clinical contexts may perform similarly in genetic testing contexts. However, the 22% of cases in which the genetics study found an effect while the overall study did not could imply that genetics might be a topic that is more easily communicated than other topics addressed in the larger reviews. Our culture’s strong belief in genetic determinism may explain why interventions whose topic is genetics may sometimes be effective while those that use similar methods on another topic are not. Looking at the effectiveness of each type of intervention on the different outcomes, all types of intervention were reported effective on one type of outcome or another, with an overall effectiveness of between 68% (decision aids) and 100% (audit and feedback; mobile phone messaging; and educational outreach to health professionals). However the low number of studies on the latter types of intervention makes useful interpretation difficult. These observations all suggest the need for more knowledge translation studies in the context of genetic testing.

Second, our results do indicate that in a significant number of trials, KT interventions such as decision aids have an effect on patient knowledge and wellbeing, while communicating DNA-based disease estimates has an effect on patient behavior. In view of current health policies in the USA, UK and Australia that promote shared decision making regarding clinical interventions [61], it may be reasonable to promote a combination of these kinds of intervention among patients and health consumers who need to make informed decisions about genetic testing. Our results indicating that decision aids had no effect on patients’ behavior-related outcomes suggest that decision aids may not be sufficient to change patient behavior in this context. Also, although costs are highly relevant data for policy makers [19], only one study reported cost analysis. Future research on KT interventions in medical genetics and genomics will need to address these gaps to better inform policy makers.

Third, more than half (17/28) of the included studies assessed interventions in the oncology context, mainly in breast and/or ovarian cancer (12/17). Hereditary breast and ovarian cancers are frequently encountered in clinical practice, and involve communicating complex information regarding cancer prevention and treatment and support for the preference-sensitive decisions faced by patients [15]. However, half of the studies (6/12) in the context of breast and ovarian cancer aimed only to increase the uptake of genetic testing, an outcome that does not provide evidence of the effectiveness of the intervention for improving the quality of the decision-making process, or even necessarily for improving overall health. A refusal to take the test could reflect a comprehensive decision-making process in which the patient’s values and preferences are fully respected. Two studies aimed to increase or decrease the uptake of genetic testing and improve behavior and wellbeing, and only four studies aimed to increase informed decisions by improving behavior and wellbeing. However, all studies (n = 5) in the context of lung cancer aimed to improve health behavior and informed decisions. Also well represented in the included trials were decisions about prenatal screening for Down syndrome, also among the most common and difficult decisions encountered in family practice [8]. Previous studies have shown that many pregnant women are ambivalent with regard to the screening and diagnosis of Down syndrome [16] and that there are unmet needs for informed decision support among patients [15, 62]. Most of the studies (5/7) in the context of fetal anomalies aimed to increase informed decisions by addressing health behavior and wellbeing.

Lastly, we observed that very few KT intervention trials had been performed with health professionals as their main target or even as one of the targets. Although the most frequent target of the KT intervention in the included studies is the patient, systematic reviews of KT interventions in other clinical areas reveal that targeting both the patient *and* the healthcare professional appears more promising in terms of effectiveness than targeting either the patient or the health professional alone [63, 64]. Therefore, our results, which focused on genetic testing already happening in the clinic, suggest that more KT intervention research targeting health professionals is needed for better translation of genetic knowledge into clinical practice. Indeed, as there are growing calls for health systems and professionals to tackle overuse of ineffective tests or treatments options [58], genetic testing contexts should not be an exception.

Our review has some limitations. First, we restricted our search strategy to the online databases of the Cochrane EPOC Review Group and the Cochrane Consumers and Communication Review Group. This search strategy and our timeline meant that we may have missed some studies. However, as we were focusing on KT interventions relevant to all decision making about genetic testing, our strategy ensured we would rapidly access a large number (n = 2473) of high-quality trials. Quality assessment of included trials showed that most were of high quality, thus reinforcing the validity of our conclusions. Second, we did not reanalyze data from the included trials and relied on the authors’ reported results. Lastly, as this was a narrative review, we did not contact authors of included trials and reported their results as published.

## Conclusions

### Implications for practice

Evidence regarding KT interventions for meeting the complex decision-making needs of patients regarding genetic testing is lagging behind the rapidly expanding knowledge about medical genetics and genomics and the increasing availability of genetic tests. In addition, the validity and/or utility of some tests are being questioned. Notwithstanding these observations, our results indicate that KT interventions related to decision making about genetic testing that target patients, such as decision aids and personalized risk communication, are more likely to be effective than not in improving knowledge, behavior and wellbeing and enabling patients and their health professionals to make enlightened decisions together. Thus both types of intervention may be relevant to the application of current health policies regarding genetic testing in many industrialized countries. Nonetheless, KT interventions targeting health professionals are still needed to foster optimal clinical practice in this context.

## Supporting Information

S1 Fig Role of genetic testing in interventions. (TIF)

S1 File PRISMA statement. (DOC)
