# Supplementary figures and images for: Improving Decision Making about Genetic Testing in the Clinic: An Overview of Effective Knowledge Translation Interventions
Source: PLoS One. 2016 Mar 3;11(3):e0150123. doi: 10.1371/journal.pone.0150123 (PMC4777394; doi:10.1371/journal.pone.0150123)

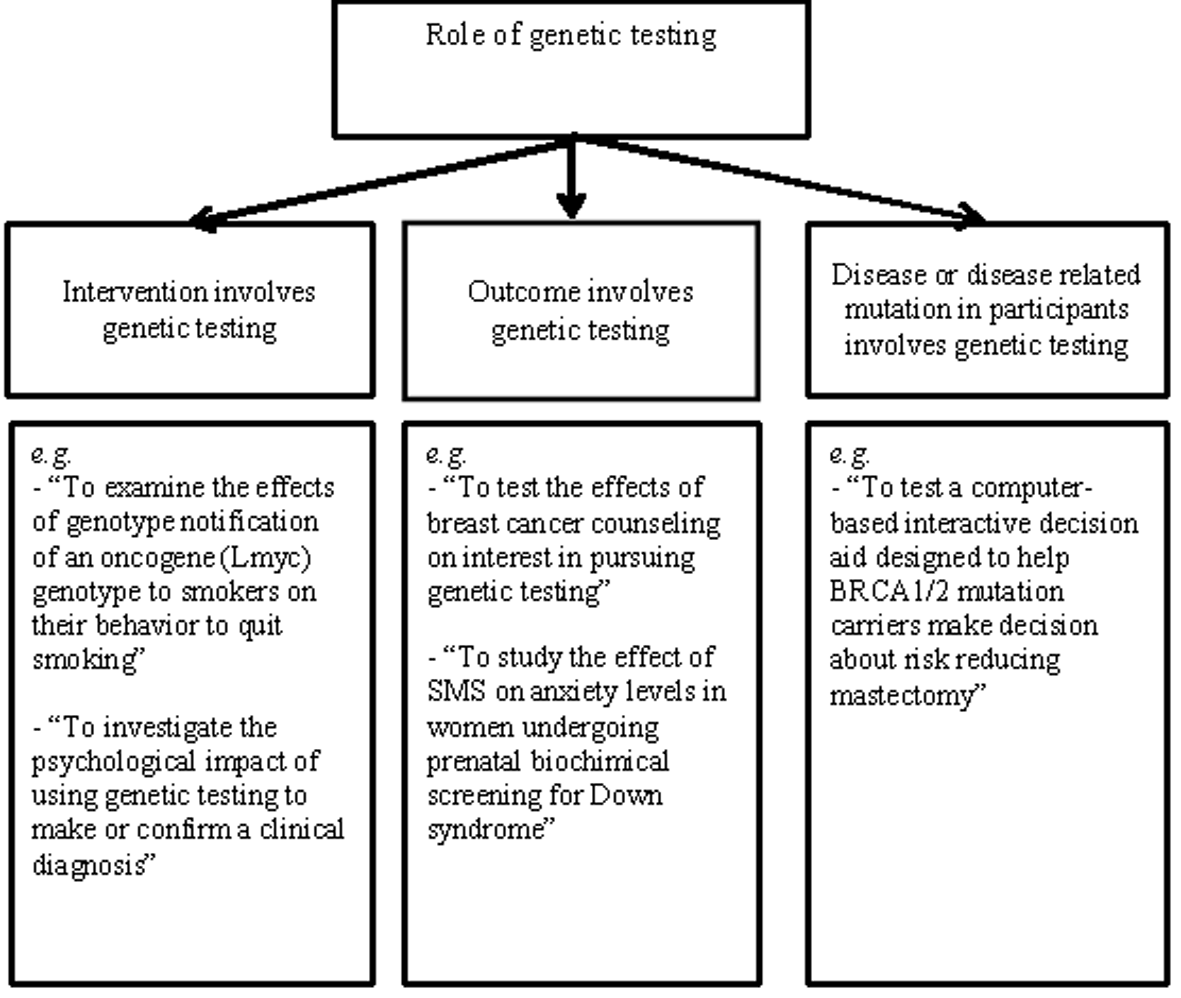

Supplement: S1 Fig — (TIF) [file pone.0150123.s001.tif]
